# An IP-10 (CXCL10)-Derived Peptide Inhibits Angiogenesis

**DOI:** 10.1371/journal.pone.0040812

**Published:** 2012-07-16

**Authors:** Cecelia C. Yates-Binder, Margaret Rodgers, Jesse Jaynes, Alan Wells, Richard J. Bodnar, Timothy Turner

**Affiliations:** 1 Tuskegee University, Center for Cancer Research, Tuskegee, Alabama, United States of America; 2 Departments of Pathology, University of Pittsburgh School of Medicine, Pittsburgh, Pennsylvania, United States of America; 3 Veterans Affairs Pittsburgh Healthcare System, Pittsburgh, Pennsylvania, United States of America; University of Leuven, Rega Institute, Belgium

## Abstract

Angiogenesis plays a critical role in processes such as organ development, wound healing, and tumor growth. It requires well-orchestrated integration of soluble and matrix factors and timely recognition of such signals to regulate this process. Previous work has shown that newly forming vessels express the chemokine receptor CXC receptor 3 (CXCR3) and, activation by its ligand IP-10 (CXCL10), both inhibits development of new vasculature and causes regression of newly formed vessels. To identify and develop new therapeutic agents to limit or reverse pathological angiogenesis, we identified a 21 amino acid fragment of IP-10, spanning the α-helical domain residues 77–98, that mimic the actions of the whole IP-10 molecule on endothelial cells. Treatment of the endothelial cells with the 22 amino acid fragment referred to as IP-10p significantly inhibited VEGF-induced endothelial motility and tube formation *in vitro*, properties critical for angiogenesis. Using a Matrigel plug assay *in vivo*, we demonstrate that IP-10p both prevented vessel formation and induced involution of nascent vessels. CXCR3 neutralizing antibody was able to block the inhibitory effects of the IP-10p, demonstrating specificity of the peptide. Inhibition of endothelial function by IP-10p was similar to that described for IP-10, secondary to CXCR3-mediated increase in cAMP production, activation of PKA inhibiting cell migration, and inhibition of VEGF-mediated m-calpain activation. IP-10p provides a novel therapeutic agent that inhibits endothelial cell function thus, allowing for the modulation of angiogenesis.

## Introduction

The formation of new blood vessels, whether by angiogenesis or vasculogenesis, is critical for several physiological processes including embryogenesis, organogenesis and vascular remodeling. Angiogenesis is regulated by a complex and interrelated system of pathways that involve various angiogenic and angiostatic factors [1], [2]. Over- or under- expression of these factors results in pathologic conditions, as noted for excessive angiogenesis in tumors, or untimely termination of angiogenesis that results in unhealed chronic wounds [3]. Researchers have sought to better understand the signaling pathways of these angiogenesis regulators to provide new therapies to modulate these and other pathological conditions.

Recent evidence demonstrates that members of the CXC chemokine family can act as either angiogenic or angiostatic factors, depending on the presence of the ELR (Glu-Leu-Arg) motif in their NH2 terminus [1]. Among this family, the chemokines (IP-9/ITAC (CXCL11) IP-10 (CXCL10), MIG (CXCL9) and CXCL4 (PF4) lack the canonical N-terminal ELR sequence [4] and bind in common to the ubiquitous CXCR3 chemokine receptor. CXCR3 has two isoforms CXCR3-A and CXCR3-B. Recent studies have shown that CXCR3 isoforms differentially regulate cell function. Activation of CXCR3-A has been shown to induce chemotaxis and proliferation in various cells types [5], [6]. Alternatively, CXCR3-B activation inhibits migration and proliferation and induces apoptosis [6], [7], [8]. In addition, IP-10 and PF4 have been reported to be angiostatic and have anti-tumor activity via its signaling through CXCR3 [7], [9], [10].

IP-10 is secreted by a diverse spectrum of cells in many tissues, and displays pleiotrophic effects in immunity, angiogenesis, and organ-specific metastases of cancer, making it a promising therapeutic target for a wide variety of diseases. To develop IP-10 as a therapeutic agent, structural details of its mechanism of action are needed to understand its role in the aforementioned pathological conditions. It has previously been determined that IP-10 consists of three anti-parallel β-sheets overlayed by an α-helix at the C-terminus [11]. The N-loop region of the β-sheets has been suggested to play a role in the binding of the protein to the receptor [12], but the domain responsible for receptor-activation is not well understood. As such, we sought to identify the functional domain of IP-10 that is responsible for CXCR3B activation and the resulting inhibition of endothelial cell function. We identified by homology modeling a 22 amino acid peptide spanning the α-helical domain of IP-10, residues 77–98, which has the ability to activate CXCR3B. We show that IP-10p is able to inhibit endothelial cell motility, vessel formation and induce vessel dissociation. In this study, we demonstrate that the α-helix domain of IP-10 is able to inhibit endothelial cell motility, vessel formation and induce vessel dissociation via direct binding and activation of the CXCR3B receptor.

## Results

### Synthetic IP-10p Binds to Endothelial Cells

**Figure 1:**
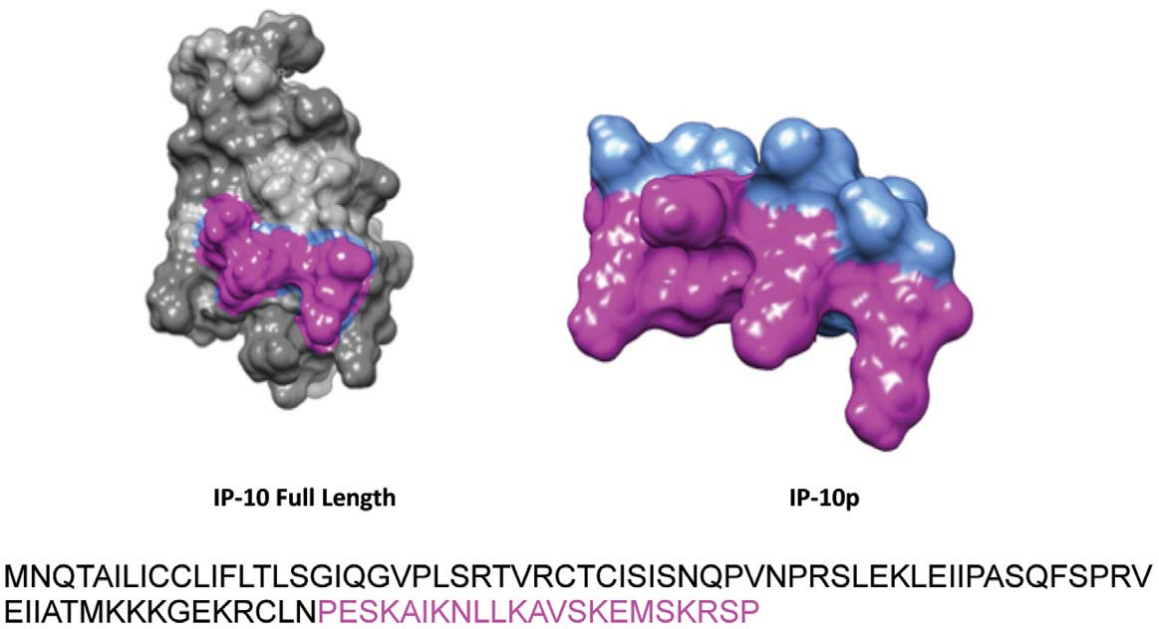
Structure model of IP-10 and IP-10p. Full Length Sequence of IP-10 with C-terminal 22 amino acids highlighted comprising IP-10p.

**Figure 2:**
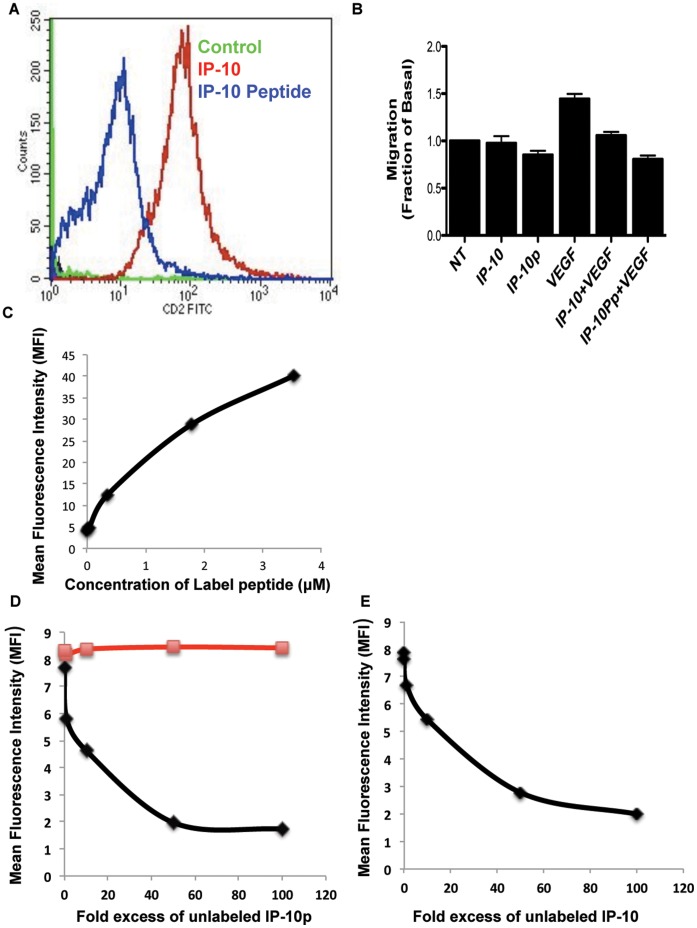
Binding of IP-10p to HMEC cells occurs via CXCR3. A) Biotin tagged IP-10 and IP-10p were incubated with cells and then probed with FITC-conjugated with streptavidin and analyzed on a BD FACSCalbur flow cytometer. Control cells were incubated with FITC-Streptavidin alone. Motility analysis shows the effects of biotinylated IP-10 (23.2 µM), IP-10p (10 µM) C) Binding of IP-10p to endothelial cells is saturable. Endothelial cells were incubated with increasing doses of IP-10p as indicated. Cells were extensively washed in PBS with FITC-Streptavidin analyzed by flow cytometry. Mean fluorescent intensities of labeled cells are plotted against the concentrations of IP-10p. D) IP-10p binds specifically to endothelial cells. Endothelial cell were incubated with 1ug/ml biotin labeled IP-10p and increasing control of unlabeled IP-10p or scrambled control at peptide. Cells were stained with FITC-Streptavidin analyzed by flow cytometry. (µg unlabeled IP-10p ν unlabeled scrambled peptide) E) IP-10 competes with IP-10p for binding. Endothelial cells were incubated with 1ug/ml biotin labeled IP-10p and IP-10. Cells were stained with FITC-Streptavidin analyzed by flow cytometry. Mean fluorescent intensities are plotted as a function of increasing quantities of competitor proteins. Data shown are of N = 6.

**Figure 3:**
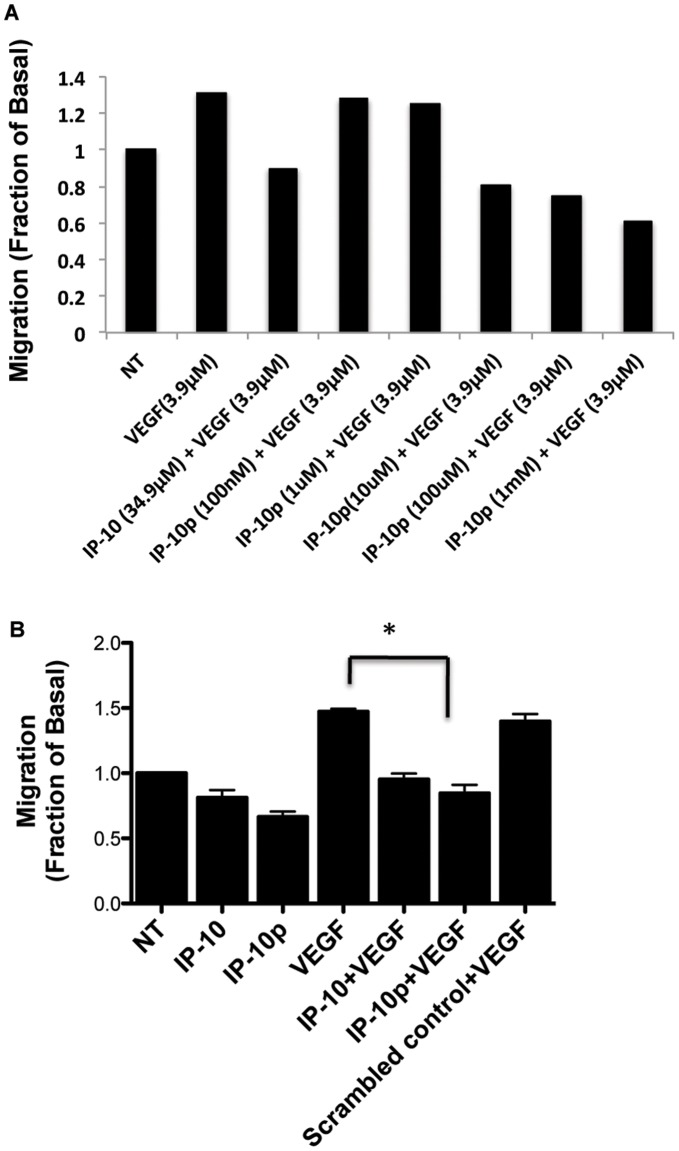
2D scratch assay of stimulated HMEC-1 cells was used to analyze migration patterns. A) The dose response used to determine the optimal concentration IP-10p (10 µM) used to compare to IP-10 (34.9 µM) B) HMEC-1 cells were grown to 80 to 85% confluence in a 12-well plate and quiesced in 0.5% dialyzed fetal bovine serum for 24 hours. A 1-mm scratch was made to the confluent of monolayer using a rubber policeman. The cells were then incubated in 0.5% dialyzed with/without IP-10 (23.2 µM), IP-10p (10 µM), VEGF (3.9 µM) or and/or scrambled control (10 µM) for 24 hours. As expected, IP-10p inhibited motility of the HMEC-1 as wells as inhibited VEGF induced motility. The results are N = 6 (average ±SEM). *P<0.05.

We were looking to identify a small peptide agonist that would activate CXCR3B. This will have a distinct advantage over the large and labile natural peptides in production and stability. To identify the active region of IP-10 that is required for the activation of CXCR3, we analyzed the protein sequence for properties similar to other known angiostatic molecules. Our initial search was based on lytic peptides, which are small basic proteins of 23–39 amino acids that have the potential to form amphipathic α-helices or partial β-pleated sheets. These small oligo peptides have been shown to interact with various membrane receptors. Using this as the modality for our analysis, we identified the α-helical region of IP-10 as a possible agonist for CXCR3 (Figure 1). The peptide identified spans the C-terminal 22 amino acid residues 77–98 that encompasses the α-helix (Accession# P02778) [13]. To determine whether IP10p binds to CXCR3 on human dermal microvascular endothelial cells (HMEC-1) [4], we analyzed the ability of Biotin-tagged IP-10 and IP10p to compete for binding to human microvascular endothelial cells HMEC-1 using flow cytometery. We tested whether IP-10p binds to HMEC-1 cells. While this data cannot determine the affinity of IP10p for CXCR3, it does show that IP10p effectively binds to endothelial cells (Figure 2A). Motility assay was performed to determine the biological effects of biotinylation activity of IP-10 and IP-10p using the optimal concentration determined in Figure 3A. Endothelial cell migration was inhibited by biotinylated IP-10 and IP-10p as expected (Figure 2B). As such, to show that IP-10p actually binds to CXCR3 we performed a competition assay with IP-10.

To perform the competition assay we first needed to determine a suboptimal amount of IP-10p that significantly labels the cell. A saturation assay was performed by incubating fixed cells with six increasing doses of biotin labeled peptide as indicated in the methods. Cells were robustly washed free of unbound labeled IP-10p and stained with FITC conjugated Strepavidin. Cells were analyzed by flow cytometry and the mean fluorescence intensity (MFI) obtained. All incubations and washes were performed at 4°C to minimize internalization. Figure 2C shows that increasing MFI was obtained with increasing concentration of biotin labeled IP-10p and saturability was observed above 4 µM (10 µg/ml) of labeled IP-10p. Based on these results all the competition assays were performed with the sub-optimal concentration of labeled IP-10p at 4 µM.

In the first competition assay, the specificity of binding of the biotin labeled IP-10p to endothelial cells was assessed by competition with increasing concentrations of unlabeled IP-10p. A fixed number of endothelial cells were co-incubated with 1 µg/ml biotin labeled IP-10p and increasing concentrations of unlabeled IP-10p as indicated on the x-axis of the graph (Figure 2D). Cell were washed and stained with Strepavidin-FITC and analyzed by flow cytometry. As shown in Figure 2C unlabeled IP-10p was able to compete away the signal demonstrating the specificity of the binding of IP-10p to endothelial cells. To verify specificity of IP-10p, an unlabeled 22 amino acid scrambled peptide failed to inhibit IP-10p binding to endothelial cells.

In the second competition assay, we determined whether IP-10p bound to the same receptor as IP-10. A fixed number of endothelial cells were co-incubated with 1ug/ml biotin labeled IP-10 peptide and increasing concentration of unlabeled IP-10 as indicated on the x-axis of the graph (Figure 2E). Cells were washed and stained with Strepavidin-FITC and analyzed by flow cytometry. Unlabeled IP-10 was able to prevent IP-10p binding to endothelial cells indicating that IP-10p binds to the same receptor on endothelial cells. As CXCR3 is the sole receptor for IP-10, these data indicate that IP-10p also binds to CXCR3.

### IP-10p Inhibits Endothelial Cell Motility

IP-10 inhibits human microvascular endothelial cells (HMEC-1) motility in the presence of angiogenic growth factors VEGF_165_ and bFGF (data not shown). [7], [14]–[16] To examine whether IP10p could mimic the inhibitory effect of the full length IP-10, HMEC-1 were analyzed using a scratch assay. Cells were tested for their ability to migrate into a denuded area over a 24-hour period in the presence or absence of VEGF_165_. Treatment with IP-10p significantly inhibited HMEC-1 cell migration in the absence or presence of VEGF (Figure 3). Figure 3A shows the dose response used to determine the optimal concentration of IP-10p used. IP-10p (10 µM) was found to inhibit endothelial cell migration to the same degree as IP-10 (Figure 3B). Scrambled control peptide was used at 10 µM and showed no such inhibition. These data demonstrate that IP-10p is functional and is at least equivalent in effect to IP-10 in cell motility inhibition.

### Endothelial Tube Formation is Inhibited by the IP-10p

**Figure 4:**
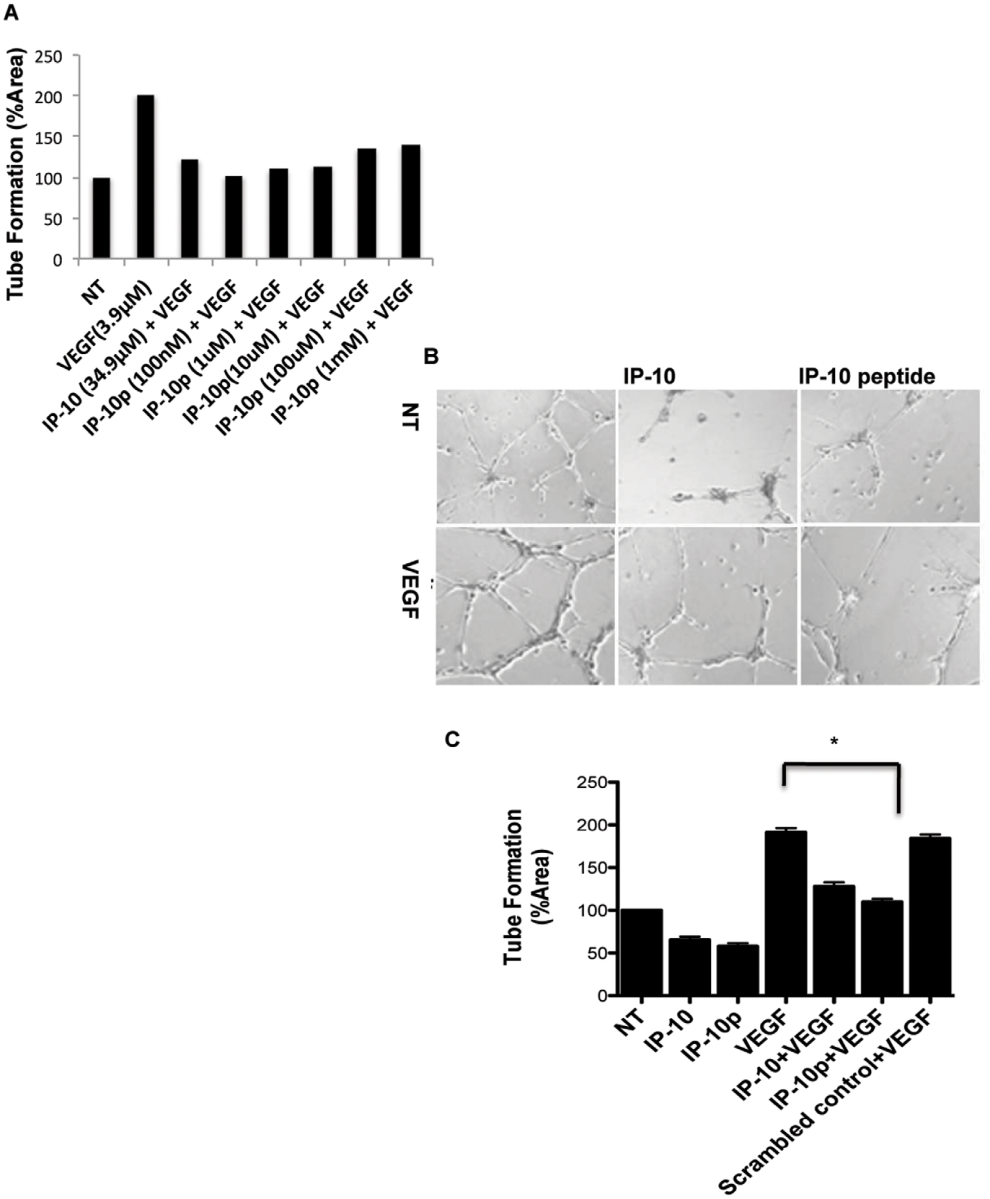
IP-10p is able to inhibit tube formation. A) The dose response used to determine the optimal concentration IP-10p (10 µM) used to compare to IP-10 (34.9 µM). B) HMEC-1 cells were grown, detached and resuspended in serum-free medium either with or without VEGF (3.9 µM), IP-10 (34.9 µM), IP-10p (10 µM) and/or scrambled control (10 µM) for 24 hours. Treated cells (1 x10^4^ cells/well) were added to 24-well culture plates coated with growth factor reduced Matrigel and incubated for 24 hours. C) Newly formed endothelial tubes in A were analyzed and quantified using MetaMorph image programming. Data shown are of N = 6 and normalized to no treatment (average ±SEM). *P<0.05. Original magnifications, 4X.

ELR-negative chemokines have been shown to limit vascularization by regulating endothelial cells ability to form tubes *in vitro* (6,7,10). To determine whether the IP-10p is able to inhibit tube formation, HMEC-1 cells were grown on growth factor reduced (GFR) Matrigel in the presence of VEGF_165_, IP-10 or IP-10p. Figure 4A shows the dose response used to determine the optimal concentration of IP-10p used. After incubation for 24 hours, the cells were able to form tubes in the presence or absence of VEGF_165_. When HMEC-1 cells were incubated with IP-10p, there was a significant reduction in tubes formed compared to scrambled control and even regardless of the presence of VEGF_165_ (Figure 4B). Quantification of tube formation demonstrates IP-10p was able to reduce tube formation slightly better than that observed for full length IP-10, in the presence of VEGF (Figure 4C).

### IP-10p Induces Tube Dissociation

**Figure 5:**
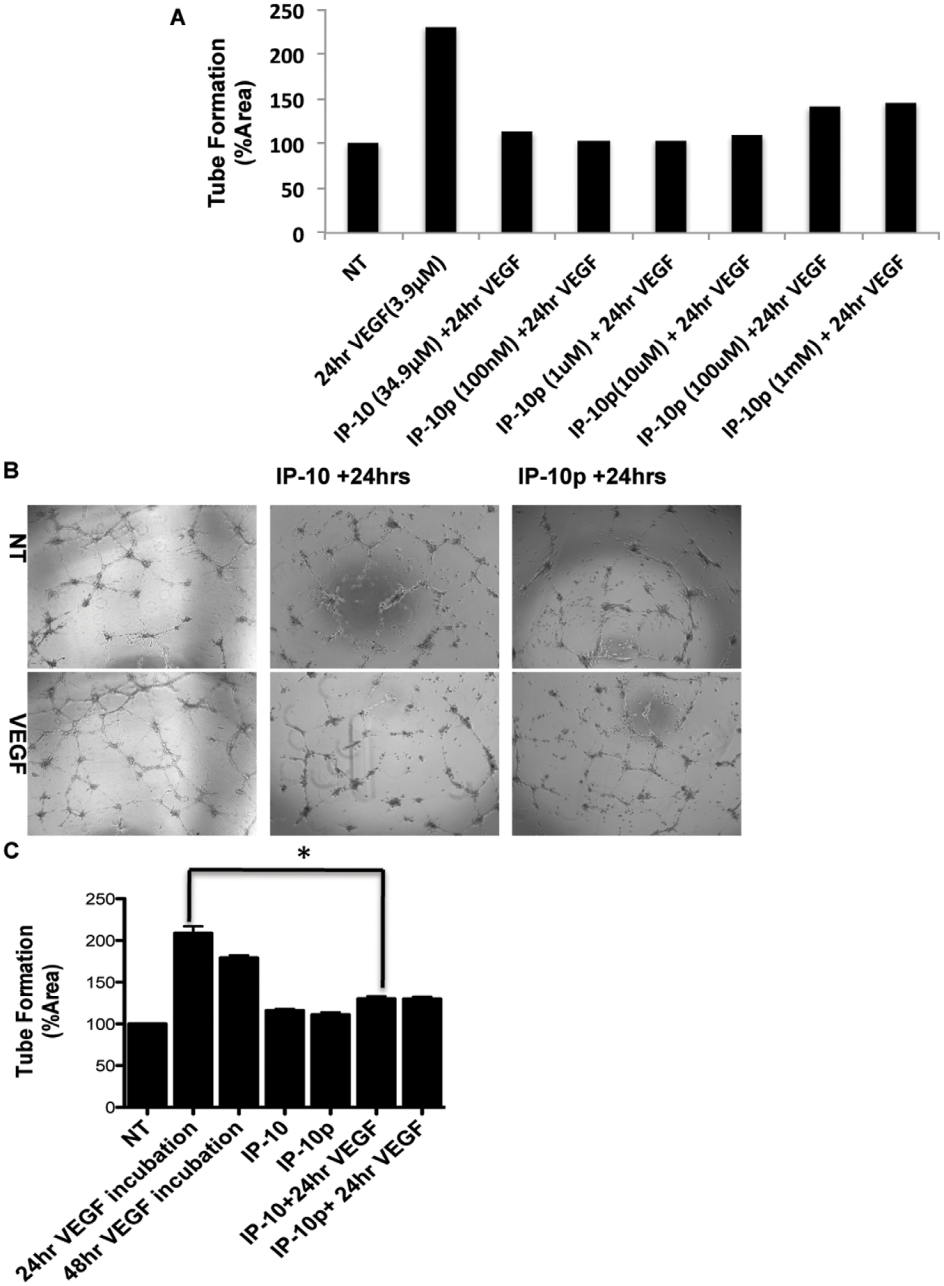
IP-10p induces dissociation of newly formed tubes. A) The dose response used to determine the optimal concentration IP-10p (10 µM) used to compare to IP-10 (34.9 µM). B) HMEC-1 cells were treated with VEGF (3.9 µM) and plated on GFR-Matrigel to form endothelial tubes. The newly formed tubes were incubated in 0.5% dialyzed FBS medium for 24 hours with VEGF (48 hours) in the presence of IP-10 (34.9 µM) (24 hrs+IP-10), or IP-10p (10 µM) (24 hrs+IP-10). C) Quantification of the endothelial tube area was determined, using MetaMorph. Data shown are of at least N = 6 and normalized to no treatment (average ±SEM). *P<0.05. Original magnifications, 4X.

IP-10 not only inhibits cells migration and tube formation, but also drives involution of nascent vessels [7], [10]. Thus, we determined whether IP-10p also induces dissociation of newly formed tubes. Using the Matrigel assay *in vitro*, HMEC-1 cells were plated on GFR-Matrigel for 24 hours to allow the formation of tubes. The medium was removed and replaced with medium containing VEGF and molar equivalent IP-10 or IP-10p then incubated for another 24 hours. After 48 hours, tubes were still present in the controls treated with VEGF alone (Figure 5). Figure 5A shows the dose response used to determine the optimal concentration IP-10p used. With IP-10p treatment, there was a significant reduction in the number of tubes observed compared to control, VEGF (Figure 5A & B). The reduction of the number of tubes was equivalent to treatment with IP-10.

### IP-10p Signal through CXCR3

**Figure 6:**
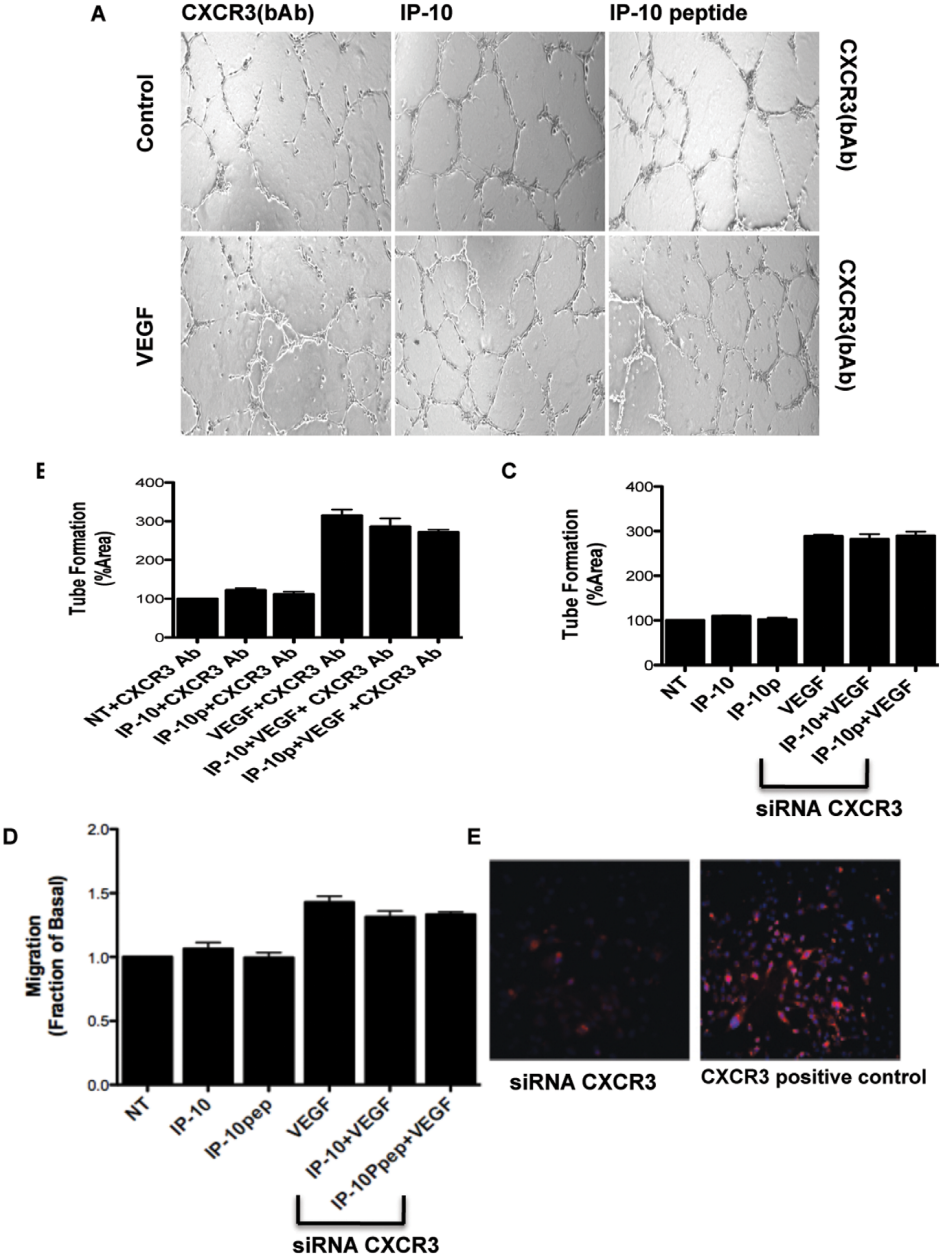
CXCR3 neutralizing antibody blocks IP-10p inhibition affects. A) HMEC-1 cells were grown, detached and resuspended in serum-free medium and pretreated with a neutralizing antibody to CXCR3 (0.5 µg/ml) 30 minutes prior to addition to VEGF (3.9 µM), IP-10 (34.9 µM), IP-10p (10 µM) and/or IgG (control). Treated cells (1 X10^4^ cells/well) were added to 24-well culture plates coated with growth factor reduce Matrigel and incubated for 24 hours. Endothelial tubes were allowed to form. B) Quantification of the endothelial tube was done using MetaMorph C) CXCR3 siRNA down regulation of CXCR3 was used on the HMEC-1 cells and incubated on GFR-Matrigel in the presence of IP-10 VEGF (3.9 µM), IP-10 (34.9 µM) and/or IP-10p (10 µM). Quantification of the endothelial cell tube density was shown using MetaMorph analysis. D) To demonstrate that the IP-10p inhibition of motility is mediated via CXCR3, a siRNA down regulation of CXCR3 was used on the HMEC-1 cells. The 2-D scratch assay was performed on the CXCR3 knockdown cells under the same condition above. Both IP-10 and IP-10p were unable to block VEGF induced motility. E) Immunofluorescence staining to verify siRNA knockdown of CXCR3. Data shown are of N = 6 and normalized to no treatment (average ±SEM). *P<0.05. Original magnifications, 4X.

The inhibitory effects of IP-10 on endothelial cells has been shown to be meditated via CXCR3 signaling. To determine if IP-10p is acting through the same signaling pathways, we used a CXCR3 neutralizing antibody and assessed the ability of IP-10p to block tube formation as shown in Figure 5. HMEC-1 cells were grown on growth factor reduced Matrigel in the presence of VEGF and molar equivalent IP-10 or IP-10p, and with the presence or absence anti-CXCR3 blocking antibody. After 24 hours on growth factor reduced Matrigel, HMEC-1 cells were able to form tubes in the presence or absence of VEGF. As expected, incubation with VEGF showed a significant enhancement in the number of tubes formed compared to the untreated cells (Figure 6A and 6B). When HMEC-1 cells were incubated with IP-10p in the presence of the anti-CXCR3 antibody, the inhibitory effect of IP-10p was reversed. IP-10p was unable to override the angiogenic signals from VEGF, and limit tube formation in the presence of VEGF when CXCR3 receptor was neutralized (Figure 6A and 6B). A similar observation was made when IP-10p was replaced with IP-10. These data strongly suggest that the IP-10p inhibitory effect on endothelial cells is CXCR3-mediated.

To further verify that the inhibitory effect of IP-10p is mediated through CXCR3, HMEC-1 were treated with CXCR3 specific siRNA and then incubated on GFR-Matrigel in the presence of IP-10p. The tube density of the siRNA-mediated knockdown cells was similar to non-specific siRNA treated cells and untreated HMEC-1 cells incubated in the absence of IP-10p (data not shown). When the endothelial cells were transfected with CXCR3 siRNA neither IP-10 nor IP-10p inhibited tube formation in the absence or presence of VEGF (Figure 6C). This CXCR3-dependence was also noted for endothelial cell migration; IP-10p did not inhibit endothelial migration of CXCR3 siRNA-treated cells into the denuded space. In comparison, IP-10p did inhibit cell migration of the CXCR3 siRNA transfected cells (Figure 6D). Knockdown of CXCR3 in these cells was verified by immunostaining for CXCR3. The CXCR3 siRNA treated cells showed a significant reduction in CXCR3 staining, where as the cells transfected with scramble siRNA (control) stain positive for CXCR3 (Figure 6E). Together these data show the inhibitory effects on both VEGF induced tube formation and cell migration are due to IP-10p mediated activation of CXCR3.

**Figure 7:**
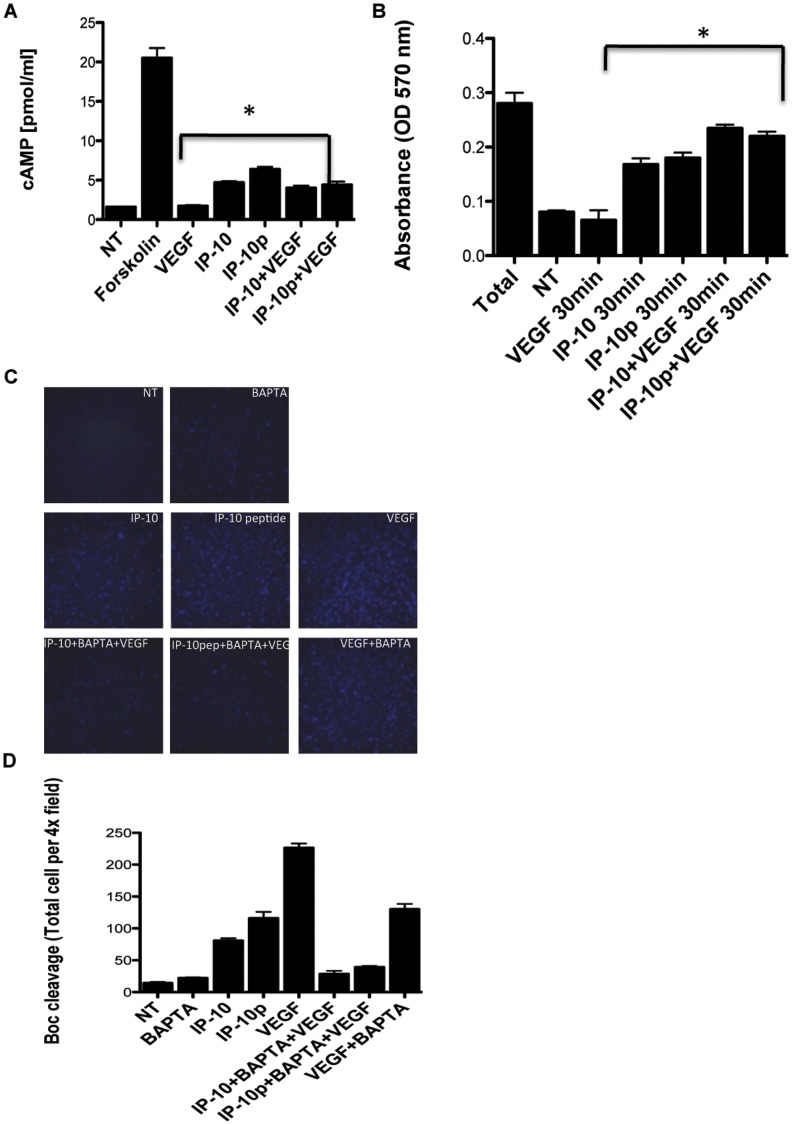
IP-10p induces cAMP activation of PKA. A) HMEC-1 cells were stimulated with 0.1% DMSO (no treatment) and/or with forskolin (25 mM), VEGF (5.2 µM), IP-10 (23.3 µM), IP-10p (10 µM) in combination. Cell lysate was analyzed for total cAMP using cAMP enzyme immunoassay kit. B) HMEC-1 were stimulated with VEGF (5.2 µM) and/or IP-10 (23.3 µM), IP-10p (10 µM) and in combination for 15, 30 and 60 minutes. The cells were lysed in a hypotonic solution containing aprotinin and PMSF. The lysate was then analyzed for PKA activity using a PKA -assay kit (Promega) and absorbance was read at 570 nm with the solubilization buffer serving as a blank. The graph shows the amount of the synthetic substrate phosphorylated by PKA. C) HMEC-1 cells were plated on gelatin-coated glass chamber slides at 1.2×10^4^ cells/well and incubated for 24 hours and then further incubated in 0.5% dialyzed fetal bovine serum for 24 hours. BAPTA AM (5 mM), or Calpain inhibitor I (CI-1, 10 mM) at 37°C for 30 minutes. The BOC-LM-CMAC (Boc) (25 µM,) was added and incubated at 37°C for 30 min, then VEGF (3.9 µM), IP-10 (23.3 µM), and IP-10p (10 µM) was added and incubated for 30 minutes at 37°C. In some experiments the addition of cAMP analogs 8-Br-cAMP (50 µM), activator of PKA, and Rp-8-Br-cAMP (250 µM), inhibitor of PKA for 20 minutes prior to the addition of Boc. Calpain activation was analyzed by fluorescence microscopy. D) Calpain activity was quantified by MetaMorph analysis. Data shown are of at least N = 9 and normalized to no treatment (average ±SEM). *P<0.05. Original magnifications, 10X.

In endothelial cells and fibroblasts, IP-10 mediated signaling is through CXCR3 and has been shown to induce cAMP activation of PKA and is required for inhibiting tube formation and motility [7], [8]. In contrast, cAMP is not increased when keratinocytes are exposed to IP-10 resulting in motogeniesis [17], [18]. As such, to determine if stimulation of endothelial cells with IP-10p induces an increase in cAMP, HMEC-1 cells were incubated with VEGF in the presence or absence IP-10 or IP-10p. Forskolin was used as a control. Cells were then assayed for total cAMP production. Treatment of HMEC-1 cells with IP-10p showed a 2-3-fold increase in cAMP production compared to untreated and VEGF treated cells (Figure 7A). When the cells were stimulated with a combination of VEGF and IP-10p, there was still a significant increase in cAMP production compared to VEGF alone. These results indicate that incubation of endothelial cells with IP-10p induces the formation of cAMP.

Protein Kinase A (PKA), a cAMP-dependent kinase, has been previously shown to be an inhibitor of endothelial cell migration and tube formation [7], [19]. Herein we sought to determine if the increase in cAMP in endothelial cells by the induction of IP-10p results in the activation of PKA. HMEC-1 cells were treated with VEGF, IP-10, IP-10p alone or in various combinations. Using a non-radioactive detection assay, total phosphorylated protein was quantified by spectrophotometry. Incubation of HMEC-1 cells with IP-10p showed a significant increase in PKA activity in comparison to VEGF and untreated cells (Figure 7B). These results suggest that IP-10p activates PKA.

In endothelial cells, the CXCR3-activation of PKA inhibits motility through inhibition of m-calpain (CAPN2) [7]. Recently, it has been established that VEGF induces m-calpain activity in endothelial cells. In endothelial cells, and in other cells μ-calpain (CAPN1) is activated secondary to a calcium flux [17] whereas m-calpain is activated, at least in part, by ERK phosphorylation on serine 50. We tested the IP-10p effect on m-calpain by using membrane permeable synthetic calpain substrate Boc-LM-CMAC. Keeping in mind that the pre-fluorescent substrate Boc-LM-CMAC can be cleaved by both m- and μ-calpain, we used BAPTA/AM, a membrane permeable calcium chelator, to distinguish between m- and μ-calpain activation in cells, as this blocks activation of μ-calpain but not m-calpain [10], [17]. Cells were treated with VEGF in the presence of IP-10 and/or IP-10p so cleavage of Boc-LM-CMAC was observed by the presence of fluorescence. When the cells were pre-incubated with BAPTA/AM, which was similarly extinguished by IP-10 and IP-10p, comparable results are observed (Figure 7C). These results were quantified to show total cells per field (Figure D). These data suggest that the IP-10p inhibits VEGF mediated m-calpain activity.

### IP-10 Peptide Inhibits the Formation of New Vessels and Causes the Dissociation of Newly Formed Vessels

**Figure 8:**
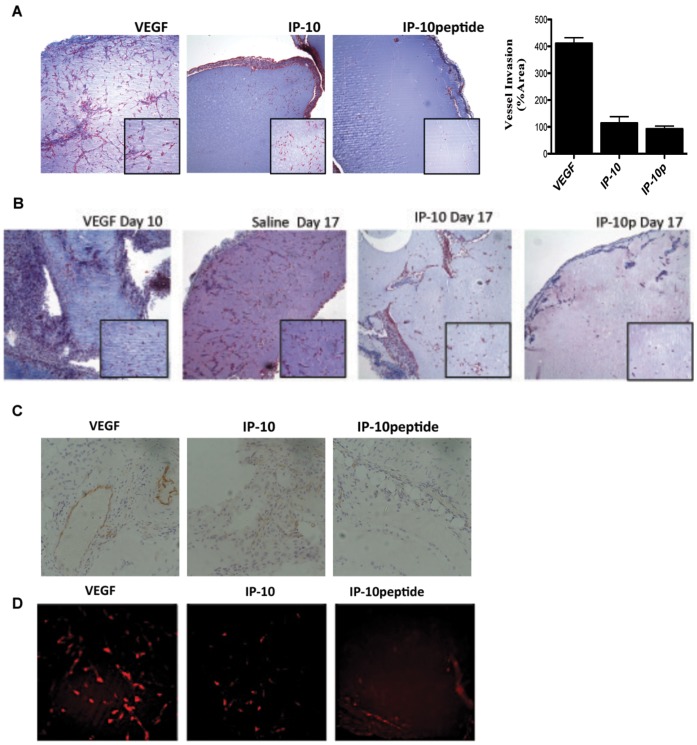
IP-10p is able to inhibit angiogenesis. A) C57BL/6J mice were subcutaneously implanted with 750 µl of Matrigel containing VEGF (23.3 µM), IP-10 (1.2 µM) and/or, of IP-10p (41 µM). Ten days post inoculation; the Matrigel plug was removed and stained with Masson Trichrome to visualize endothelial infiltration. Vessels were quantified by counting vessel infiltration in to the plug on day 10. B) To verify that IP-10p is able to inhibit regression of newly formed vessel *in vivo* GFR- Matrigel supplemented with VEGF (10.4 µM) was injected into both the left and right side groin of mice. After 10 days the left side Matrigel plug was removed (control, *n* = 6) to show vessel invasion. The remaining right side Matrigel plug in the mice was injected with either IP-10 (1.2 µM) and/or, IP-10p (41 µM) at days 10 and 12. Day 17, the Matrigel plugs were removed and stained with Masson’s Trichrome. C) To validate the invasion of endothelial cells plugs were stained with CD31. IP-10p treated plugs showed a significant regression of vessels compared to the saline controls. D) Vasculature is a marker of vessel maturation Desmin to validate mature vessels. Images shown represent N = 8 Original magnifications, 10X and/or 40X.

Thus far we have demonstrated that IP-10p is able to inhibit endothelial tube formation and induce the dissociation of formed tubes *in vitro*. To determine whether IP-10p is able to inhibit vessel growth, an *in vivo* Matrigel assay was used to determine whether IP-10p is able to inhibit angiogenesis. GFR-Matrigel supplemented with VEGF_165_ only was injected into one side of the inguinal region of mice. The other side was injected with Matrigel containing VEGF and IP-10p. The matrigel was incubated for 10 days to allow vessel invasion into the Matrigel. The Matrigel plug was removed and examined histologically using Masson’s trichrome staining. The staining showed that while VEGF induced endothelial invasion and formation of vessels, IP-10p inhibited this angiogenesis in the presence of VEGF (Figure 8A). These vessels were quantified and revealed the IP-10p inhibition. These results indicate that IP-10p has the ability to inhibit VEGF-induced vessel formation. In addition, it has been previously shown that IP-10 can mediate vessel regression of newly formed vessels *in vivo* [10]. To test whether the IP-10p could mediate the same regression in an *in vivo* environment, Matrigel containing VEGF was injected into the subcutaneous space of mice. On day 10 vessels were observed in the matrigel (Figure 8B, VEGF Day 10). On days 10 and 12 one side of the inguinal region was inoculated with saline and the other with IP-10p. At day 17 post Matrigel injections, the implanted Matrigel plugs were removed and analyzed for vessel formation. Our findings show that IP-10p treatment causes the dissociation of newly formed vessels (Figure 8B, IP-10p day 17). The vessel dissociation incurred by IP-10p was similar to that observed with IP-10 (Figure 8B, IP-10p day 17 and IP-10 day 17). Vessel dissociation was not due to a lack of trophic factors to the matrigel as the day 17 saline-treated Matrigel showed an increase in vascular density compared to day 10 (Figure 8B). The plugs were stained with CD31 to validate endothelial cells immigration into the plug (Figure 8C). Additionally, the plugs were stained with desmin marker of vessel maturation. The staining shows less mature vessels in the presence of IP-10p. These data indicate that IP-10p is able to promote the dissociation of newly formed vessels.

## Discussion

Angiogenesis is modulated by chemokines secondary to their influence on endothelial cell migration, proliferation, and survival [20]. Thus, harnessing the effects of a key chemokine, would provide an entrée to control of pathological vessel growth. IP-10 (CXCL10), a chemokine secreted by a diverse range of tissues and highly expressed in a wide variety of diseases, is known to be angiogenic. Previously, we have reported that by binding to CXCR3 on endothelial cells IP-10 can limit new vessel growth by inhibiting endothelial cell migration [7], and induce involution of new vessels by triggering endothelial cell anoikis [10]. It needs to be noted that IP-10 does not block the motility of keratinocytes, but rather increases their motility [15]. This is explained by the diverse modulation occurring via the activation of two separate downstream pathways from CXCR3 [7], [21], [22]. However, what is significant is that the effect on endothelial cells and angiogenesis is consistent *in vitro* and *in vivo*, and thus can be utilized to limit new vessel growth.

CXCR3 is a seven transmembrane G-protein receptor that exist as two variants called CXCR3-A and CXCR3-B. It has been shown that CXCR3-A promotes chemotaxis and cell proliferation while CXCR3-B stimulates signals for growth inhibition [23]. In recent reports, CXCR3 signaling results in chemotactic activation of keratinocytes via a PLCβ pathway that induces μ-calpain activation, which is mediated by calcium influx. Whereas in endothelial cells, chemotaxis is blocked via the inhibition of m-calpain by a cAMP-PKA mediated pathway [7]. Therefore, it is suggested that the regulation of these very different cellular responses is due to CXCR3-A/B binding of chemokines.

As structure dictates function, the structural details of IP-10s are fundamental to understanding IP-10-mediated processes via CXCR3, and to develop CXCR3-targeted therapeutics. Herein we sought to, and now report, epitopes that are at least in part responsible for the inhibition of chemotaxis and calcium influx in endothelial cells. Prototypical of CXC chemokine topology IP-10 secondary structure consists of an elongated N terminus that precedes the first cysteine, followed by a region of the structure which is between the second cysteine and the 3_10_ helix known as the N loop. This single-turn 3_10_ helix is succeeded by three β-strands and a C-terminal α-helix [11], [24]. Previously peptides have been developed that correspond with the β-strands, particularly β-strand 1, which have been identified as the binding site responsible for receptor-mediated chemotaxis and calcium flux [11]. To determine the region of IP-10 that is responsible for the inhibitory function in endothelial cells and results in an antiangiogenic biological function, we synthesized a peptide corresponding to the C-terminal α-helix region of IP-10 (IP-10p).

In this study, we investigated the antiangiogenic activities of IP-10p. We provide evidence that the α-helix region of IP-10 (IP-10p) is able to inhibit angiogenesis and cause the regression of newly formed vessels. Our results show that IP-10p binds and signals via the CXCR3 receptor. Further investigation is necessary to address the question of the affinity by which IP-10p binds to CXCR3. However, IP-10p stimulation of microvascular endothelial cells promotes the activation of PKA by increasing levels of cAMP resulting in the inhibition of endothelial cell migration to a degree comparable to the full length IP-10. It has been well established that m-calpain induced motility is growth factor mediated [16]. Vascular endothelial growth factor (VEGF) stimulation of endothelial cell motility, tube formation *in vitro*, and the growth of newly formed vessel formation *in vivo* are mediated by its activation of m-calpain via PKA activation. To determine the ability of IP-10p to block the effects of VEGF induced m-calpain activity, we analyzed its ability to activate PKA. Treatment of endothelial cells with the IP-10p resulted in high levels of cAMP, thus stimulating PKA, an inhibitor of endothelial function. This novel PKA mediated pathway induced by IP-10 signaling has been shown to regulate angiogenesis by stalling endothelial cell migration [7]. Our data shows that IP-10p is able to signal in the same fashion, which results in comparable inhibition. These results were observed in our *in vitro* system and *in vivo* model. Most importantly, IP-10p, similarly to the native chemokine, was able to induce involution of nascent vessels. Furthermore, our current studies show that IP-10p exerts a dominant affect on VEGF and other pro-angiogenic factors. Though these are preliminary studies requiring validation in models of pathological angiogenesis, our data provides strong evidence for the novelty of IP-10p and its ability to be an antiangiogenic inhibitor by controlling key components in vascular regression.

In summary, the data obtained *in vitro* and *in vivo* have demonstrated the efficacy of using IP-10p as a possible therapeutic target for numerous pathological conditions that results in uncontrollable angiogenesis. While containing only 22 amino acids rather than the full 98 (accession P02778), IP-10p is biologically comparable to the full length IP-10. More in-depth studies are necessary to further define the underlying molecular mechanism(s) through which IP-10p exerts its antiangiogenic activity. However, IP-10p is a promising candidate as an antiangiogenic treatment because of its small size and inhibitory functions at low doses. Equally important, this small active fragment may be useful in increasing bioavailability and enhancing therapeutic value through its combined use with engineered agents or in nanotechnology-directed therapies.

## Materials and Methods

### Peptide

The peptide was synthesized using fMoc chemistry by Polypeptide Laboratories in San Diego, CA. The IP-10 peptide purification was conducted utilizing a Beckman HPLC fitted with a YMC C18, 4.6×250 mm column at 60°C. The solvent system was: A = 0.1%TFA/H20, B = 0.1%TFA/ACN at a flow rate of 1 ml/min. Purity was determined using a LC/MS Finnigan MAT-LC/Q. This peptide was chemically synthesized and purified by HPLC, and the purity, relative mass, and sequence was confirmed (data not shown). All peptides used where diluted in culture medium to neutralize the TFA before use.

### Cell Culture

Immortalized human microvascular cell line (HMEC-1; Passages 21–25) was used in all experiments. The HMEC-1 line was obtained from the Center for Disease Control (Atlanta, GA) and grown in 10% FBS-MDCB 131 medium (Gibco, Gaithersburgh, MD) supplemented with 10 mM L-Glutamate (Gibco), 1 ng/ml EGF (BD Biosciences, Bedford, MA), 1 µg/ml hydrocortisone (Sigma, St. Louis, MO). Down regulation of CXCR3 using siRNA: HMEC-1 cells were plated at 5.5×10^5^ cells/60 mm dish and incubated overnight. The cells were 90–95% confluent. Cells were transfected with 4 µM siRNA (2 µM Sigma, 2 µM Santa Cruz) using Dharamacon #4 transfection reagent. The cells were incubated for 24 hours, then placed in EGM-2MV medium for 48 hours and subsequently transfected as indicated above. After 48 hours of incubation in EGM-2MV medium, the cells were then used for experimentation.

### Biotin Labeling, Binding and Competition Assay

Sulfo-NHS-LC-Biotin (Thermo Scientific 21327) was prepared to result in a 10 mM solution of Biotin. IP-10, IP-10p or anti-CXCR3 neutralizing antibody was dissolved in PBS and incubated with the biotin for 2 hours according to manufacturer’s protocol. IP10p contains 5 lysines while IP-10 has 10 lysines within its amino acid sequence and so there is a *potential* for a 2-fold difference in bound biotin. However, measurement of bound biotin as described in the manufacturer’s protocol revealed that only 2 biotin were bound. For binding and competition cells were suspended in PBS and Biotin label peptide or protein was added for 30 minutes. Cells were then washed to remove unbound peptide/protein and incubated with streptavidin conjugated with FITC, re-washed and analyzed on a BD FACSCalbur flow cytometer.

Cell signaling assays were carried out using HMEC-1 FITC labeled peptide described above. Cells were analyzed by flow cytometry to obtain the mean fluorescence intensity. Flow cytometry was performed on a BD FACSCalbur flow cytometer.

### Motility Assay

Cell migration was performed by plating HMEC-1 cells at 2.5×10^5^ cells/well in 12 well culture plates in complete growth medium and incubated for 24 hours at 37° in 5% CO_2_. The cells were washed one time with PBS and then incubated in 0.5% dialyzed MDCB 131 medium for 24 hours at 37°C in 5% CO_2_. The monolayer was scraped with a rubber policeman making a 1 mm wide denuded area. The cells were then stimulated with or without IP-10 (34.9 µM), IP-10p (10 µM), VEGF (3.9 µM), anti-CXCR3 neutralizing antibody and in combination for 24 hours at 37°C in 5% CO_2_. Images were taken at time zero and 24 hours, and the relative distance traveled by the cells into the acellular area was determined using MetaMorph.

### Tube Formation Assay

Growth factor reduced (GFR) Matrigel 10 µl/well (in 15-well μ-angiogenesis slides, ibidi, Germany), was incubated for 15 minutes at 37°C. HMEC-1 cells (10,000 cells/well) were resuspended in 0.5% FBS MDCB131 medium containing VEGF (3.9 µM), IP-10 (34.9 µM) and IP-10p (10 µM) and or anti-CXCR3 neutralizing antibody as described in the figure legend. The cells were plated on the Matrigel and incubated for 24 hours at 37°C to allow tube formation. For the tube dissociation assay, HMEC-1 cells were resuspended in 0.5% FBS MCDB131 medium containing VEGF (3.9 µM) and incubated on GFR-Matrigel for 24 hours at 37°C to allow tube formation. The medium was removed and replaced with 0.5% FBS MCDB131 medium containing VEGF (3.9 µM), IP-10 (34.9 µM) and IP-10p (10 µM) as indicated in the figure legend then further incubated for 24 hours at 37°C in 5% CO_2_. The cells were then imaged for tube formation using an Olympus IX70 microscope equipped with a Hammastu camera using MetaView™ software (Universal Imaging Corporation, Downington, PA). Analysis of tube formation was performed using MetaMorph (Universal Imaging Corporation, Downington, PA); and the results are shown as a percent of the no-treatment control. Quantification of the tubes was performed by taking six 4x images of each chamber, the tube formation was assessed by area of each image and was analyzed by MetaMorph and averaged together. The average tube formation is represented at a percentage of the no treatment control (7).

### cAMP Assay

HMEC-1 were plated in 10 mm culture dishes and grown to confluency in complete growth medium. The cells were then incubated in serum-reduced medium (0.5% dialyzed FBS for HMEC-1 cells) for 24 hours at 37°C. The cells were then washed once with PBS and incubated in serum-free medium. The cells were then stimulated with forskolin (25 µM), VEGF (5.2 µM), IP-10 (23.3 µM), IP-10p (10 µM) alone or in various combinations. The medium was removed, ice cold 80% ethanol was added, and the cells incubated on ice for 15 minutes. The extracts were evaluated and quantified by using cAMP enzyme immunoassay kit (Sigma, St. Louis, MO). The assay was performed according to manufacturer’s protocol.

### PKA Assay

HMEC-1 cells were grown in complete growth medium then further incubated in 0.5% dialyzed FBS MCDB 131 medium for 24 hours. The cells were then stimulated with VEGF (5.2 µM) and/or IP-10 (23.3 µM), IP-10p (10 µM) and in combination for 15, 30 and 60 minutes. The cells were lysed with an ice-cold hypotonic solution (50 mM Tris pH 7.4, 1 mM EDTA, 10 mg/ml aprotinin and 1 mM PMSF). The cells were incubated on ice for 30 minutes then centrifuged to remove the cell membranes. PKA activity was measured using a cAMP dependent protein kinase kit Non-Radioactive Detection kit (Promega). The assay was performed according to manufacturer’s protocol.

### Calpain Activation Assay

8-well chamber slides were coated with 0.1% gelatin for 24 hours at 4°C. The gelatin was removed and HMEC-1were plated at 12,000 cells/well then incubated in 10% FBS-EBM-EC medium for 24 hours. The medium was removed and the cells were further incubated for 12 hours with 0.5% dialyzed FBS-EBM medium. The cells were incubated with either BAPTA AM (5 µM), or Calpain inhibitor I (CI-1, 10 µM) at 37°C for 30 minutes. BOC-LM-CMAC (Boc) (25 µM, Molecular Probes) was added and incubated at 37°C for 30 minutes, then VEGF (3.9 µM), IP-10 (23.3 µM), and IP-10p (10 µM) was added and incubated for 30 minutes at 37°C. In some experiments, the addition of cAMP analogs 8-Br-cAMP (50 µM), activator of PKA, and Rp-8-Br-cAMP (250 µM), inhibitor of PKA for 20 minutes prior to the addition of Boc. The cells were then placed under a coverslip and analyzed for cleavage of Boc by fluorescence microscopy. Quantification of positive cells was performed by counting the total number of cells and the fluorescent cells in a 4× image using MetaMorph. The absence of fluorescent cells in the no treatment served as a control. Digital images were captured using a Spot Flex digital camera and Spot software.

### siRNA Transfection

CXCR3 siRNA transfection was performed as previously described. HMEC-1 cells were plated in a 6-well tissue culture plate at 4.0×10^5^ cells/well in complete MCDB131 medium and incubated overnight. The cells were ∼75% confluent. The medium was removed and replaced with serum free Opti-Mem and incubated for 30 minutes. Dharmafect #4 was added to Opti-Mem to a final concentration of 2.5% (final volume 200 µl) then incubated for 5 minutes at room temp. In parallel, CXCR3 siRNA was diluted in Opti-Mem to a final concentration of 200 mM (final volume 200 µl) then incubated for 5 minutes at room temp. CXCR3 siRNA was a pool of equal concentration from Sigma and Santa Cruz. The Dharmafect solution was added to the CXCR3 siRNA pool and incubated for 20 minutes at room temp. The Dharmafect/siRNA solution was diluted with 1.6 ml of complete MCDB131 medium, then added to a well and incubated for 6 hours at 37°C with 5% CO_2_. The medium was removed and replaced with complete MCDB131 medium. Cells were incubated for 48 hours at 37°C with 5% CO_2_. After 48 hours the cells were transfected a second time as indicated above. The cells were incubated for 36 hours in complete MCDB131 medium at 37°C with 5% CO_2_ then detached for use. Staining for CXCR3 was using to detect expression.

### In vivo Matrigel Plug Assay

Vessel regression *in vivo* was determined using the method previously described (7). In brief, Matrigel (500 µl) with VEGF was injected into the groin area near the dorsal midline of 12-month-old C57B1/6 female mice. The mice were injected with 50 µl of saline (control), 1.2 µM of IP-10 and/or 41 µM of IP-10p. On day 10 and 12 post Matrigel inoculation, the mice were injected in the inguinal region. One side received saline, the other an equal volume of IP-10 or IP-10p. Matrigel plugs were removed on day 10 (control for vessel invasion) and day 17 and either paraffin embedded or flash frozen. Plugs were histologically analyzed for vessels by Masson’s Trichrome. CD31 and Desmin (Abcam) were used for staining of Matrigel plugs. Quantification was performed MetaMorph and Image J software.

### Statistical Analyses

All quantitative assays were performed at least six times each in triplicate experiments. All animal assays were performed on a minimum of six mice. Results are expressed as mean ± SEM per specimen or experiment because all individual assay measurements were performed in replicate. Statistical differences between groups were determined by using a two-tailed Student’s t-test. Paired analyses were performed between all groups. Significance is deemed at P<0.05.

## References

[1] Strieter RM, Burdick MD, Mestas J, Gomperts B, Keane MP (2006). Cancer CXC chemokine networks and tumour angiogenesis.. Eur J Cancer.

[2] Dimberg A (2010). Chemokines in angiogenesis.. Curr Top Microbiol Immunol.

[3] Balestrieri ML, Balestrieri A, Mancini FP, Napoli C (2008). Understanding the immunoangiostatic CXC chemokine network.. Cardiovasc Res.

[4] Godessart N, Kunkel SL (2001). Chemokines in autoimmune disease.. Curr Opin Immunol.

[5] Kelsen SG, Aksoy MO, Yang Y, Shahabuddin S, Litvin J (2004). The chemokine receptor CXCR3 and its splice variant are expressed in human airway epithelial cells.. Am J Physiol Lung Cell Mol Physiol.

[6] Lasagni L, Francalanci M, Annunziato F, Lazzeri E, Giannini S (2003). An alternatively spliced variant of CXCR3 mediates the inhibition of endothelial cell growth induced by IP-10, Mig, and I-TAC, and acts as functional receptor for platelet factor 4.. J Exp Med.

[7] Bodnar RJ, Yates CC, Wells A (2006). IP-10 blocks vascular endothelial growth factor-induced endothelial cell motility and tube formation via inhibition of calpain.. Circ Res.

[8] Shiraha H, Glading A, Chou J, Jia Z, Wells A (2002). Activation of m-calpain (calpain II) by epidermal growth factor is limited by protein kinase A phosphorylation of m-calpain.. Mol Cell Biol.

[9] Addison CL, Daniel TO, Burdick MD, Liu H, Ehlert JE (2009). The CXC chemokine receptor 2, CXCR2, is the putative receptor for ELR+ CXC chemokine-induced angiogenic activity.. J Immunol.

[10] Bodnar RJ, Yates CC, Rodgers ME, Du X, Wells A (2009). IP-10 induces dissociation of newly formed blood vessels.. J Cell Sci.

[11] Swaminathan GJ, Holloway DE, Colvin RA, Campanella GK, Papageorgiou AC (2003). Crystal structures of oligomeric forms of the IP-10/CXCL10 chemokine.. Structure.

[12] Clark-Lewis I, Mattioli I, Gong JH, Loetscher P (2003). Structure-function relationship between the human chemokine receptor CXCR3 and its ligands.. J Biol Chem.

[13] Luster AD, Unkeless JC, Ravetch JV (1985). Gamma-interferon transcriptionally regulates an early-response gene containing homology to platelet proteins.. Nature.

[14] Ades EW, Candal FJ, Swerlick RA, George VG, Summers S (1992). HMEC-1: establishment of an immortalized human microvascular endothelial cell line. J Invest Dermatol..

[15] Yates CC, Whaley D, Kulasekeran P, Hancock WW, Lu B (2007). Delayed and deficient dermal maturation in mice lacking the CXCR3 ELR-negative CXC chemokine receptor.. Am J Pathol.

[16] Satish L, Yager D, Wells A (2003). Glu-Leu-Arg-negative CXC chemokine interferon gamma inducible protein-9 as a mediator of epidermal-dermal communication during wound repair.. J Invest Dermatol.

[17] Satish L, Blair HC, Glading A, Wells A (2005). Interferon-inducible protein 9 (CXCL11)-induced cell motility in keratinocytes requires calcium flux-dependent activation of mu-calpain.. Mol Cell Biol.

[18] Ohta K, Shigeishi H, Taki M, Nishi H, Higashikawa K (2008). Regulation of CXCL9/10/11 in oral keratinocytes and fibroblasts.. J Dent Res.

[19] Kim S, Bakre M, Yin H, Varner JA (2002). Inhibition of endothelial cell survival and angiogenesis by protein kinase A. J Clin Invest.

[20] Dimberg A (2010). Chemokines in angiogenesis.. Curr Top Microbiol Immunol.

[21] Yates CC, Whaley D, Y-Chen A, Kulesekaran P, Hebda PA (2008). ELR-negative CXC chemokine CXCL11 (IP-9/I-TAC) facilitates dermal and epidermal maturation during wound repair.. Am J Pathol.

[22] Yates CC, Whaley D, Hooda S, Hebda PA, Bodnar RJ (2009). Delayed reepithelialization and basement membrane regeneration after wounding in mice lacking CXCR3. Wound Repair Regen..

[23] Aidoudi S, Bikfalvi A (2010). Interaction of PF4 (CXCL4) with the vasculature: A role in atherosclerosis and angiogenesis.. Thromb Haemost.

[24] Jouan V, Canron X, Alemany M, Caen JP, Quentin G (1999). Inhibition of in vitro angiogenesis by platelet factor-4-derived peptides and mechanism of action.. Blood 1.

